# IgG and IgM antibody formation to spike and nucleocapsid proteins in COVID-19 characterized by multiplex immunoblot assays

**DOI:** 10.1186/s12879-021-06031-9

**Published:** 2021-04-07

**Authors:** Jyotsna Shah, Song Liu, Hari-Hara Potula, Prerna Bhargava, Iris Cruz, Denise Force, Ammar Bazerbashi, Ranjan Ramasamy

**Affiliations:** 1 IGeneX Inc, 556 Gibraltar Drive, Milpitas, CA 95035 USA; 2 ID-FISH Technology Inc, 556 Gibraltar Drive, Milpitas, CA 95035 USA; 3 Medical Art Center, 950 Route 35, Middletown, NJ 07748 USA

**Keywords:** COVID-19, Line immunoblot assay, Multiplex assay, Receptor-binding domain, SARS-CoV-2, Serological diagnosis, Spike protein, Nucleocapsid protein

## Abstract

**Background:**

Rapid and simple serological assays for characterizing antibody responses are important in the current COVID-19 pandemic caused by SARS-CoV-2. Multiplex immunoblot (IB) assays termed COVID-19 IB assays were developed for detecting IgG and IgM antibodies to SARS-CoV-2 virus proteins in COVID-19 patients.

**Methods:**

Recombinant nucleocapsid protein and the S1, S2 and receptor binding domain (RBD) of the spike protein of SARS-CoV-2 were used as target antigens in the COVID-19 IBs. Specificity of the IB assay was established with 231 sera from persons with allergy, unrelated viral infections, autoimmune conditions and suspected tick-borne diseases, and 32 goat antisera to human influenza proteins. IgG and IgM COVID-19 IBs assays were performed on 84 sera obtained at different times after a positive RT-qPCR test from 37 COVID-19 patients with mild symptoms.

**Results:**

Criteria for determining overall IgG and IgM antibody positivity using the four SARS-CoV-2 proteins were developed by optimizing specificity and sensitivity in the COVID-19 IgG and IgM IB assays. The estimated sensitivities and specificities of the COVID-19 IgG and IgM IBs for IgG and IgM antibodies individually or for either IgG or IgM antibodies meet the US recommendations for laboratory serological diagnostic tests. The proportion of IgM-positive sera from the COVID-19 patients following an RT-qPCR positive test was maximal at 83% before 10 days and decreased to 0% after 100 days, while the proportions of IgG-positive sera tended to plateau between days 11 and 65 at 78–100% and fall to 44% after 100 days. Detection of either IgG or IgM antibodies was better than IgG or IgM alone for assessing seroconversion in COVID-19. Both IgG and IgM antibodies detected RBD less frequently than S1, S2 and N proteins.

**Conclusions:**

The multiplex COVID-19 IB assays offer many advantages for simultaneously evaluating antibody responses to different SARS-CoV-2 proteins in COVID-19 patients.

**Supplementary Information:**

The online version contains supplementary material available at 10.1186/s12879-021-06031-9.

## Background

Coronavirus disease 2019 (COVID-19) caused by the severe acute respiratory syndrome coronavirus 2 (SARS-CoV-2), first detected in Wuhan, China in December 2019 has become a global pandemic with approximately 108.2 million infections and 2.4 million deaths worldwide on 13 February 2021 [1]. Infection is most commonly diagnosed by nucleic acid amplification tests (NAATs), often RT-qPCR, performed on nasopharyngeal and mid-turbinate swabs [2]. Detection of antibodies to SARS-CoV-2 is however important for several purposes including: (i) confirming present or past infection, (ii) evaluating patients with negative NAATs who show characteristic COVID-19 symptoms, (iii) sero-epidemiological studies on COVID-19, (iv) assessing the development of antibody-mediated protective immunity, and (v) investigating immune response and immunopathology in COVID-19 [3, 4]. The spike (S) and nucleocapsid (N) proteins of SARS-CoV-2 are commonly used target antigens in COVID-19 serological assays. The S protein is exposed on the outside of the virus membrane while N encapsulates viral RNA within the membrane envelope. S is composed of a N-terminal S1 region containing a receptor binding domain (RBD) which binds to the angiotensin-converting enzyme 2 receptor on host cells, and a C-terminal S2 region that subsequently mediates fusion between the viral and host cell membranes to allow the entry of viral RNA into the cell [5].

Different platforms are available for serological testing in COVID-19. Lateral flow immunoassays are common point of care serological tests and produce results in <30mins. Enzyme Immunoassay (EIA) is the most frequently used serological method in clinical laboratories [3, 4]. EIAs usually measure antibodies to single antigens in a clinical laboratory over a period of several hours, and have been important for assessing antibody responses in COVID-19 [6, 7] and COVID-19 vaccine trials [8].

We describe here for the first time the use of recombinant S1, S2, RBD and N proteins as antigens in multiplex line immunoblots (IBs) to detect antibodies in COVID-19 patients. The COVID-19 IgG and IgM IB assays detect IgG and IgM antibodies to S1, S2, RBD and N proteins in less than 3 h.

## Methods

### Human sera and goat antisera for assessing the specificity of COVID-19 IBs

A total of 231 pre-COVID-19 pandemic human sera expected to be negative for SARS-CoV-2 antibodies were obtained from the College of American Pathologists, New York State Department of Health, New York Biologics (Southampton, NY), National Institutes of Health (Bethesda, MD), BEI Resources (Manassas, VA) and IGeneX (Table 1). The IGeneX samples were left over sera that would otherwise have been discarded, and originally received for routine testing for tick-borne diseases, while the other human sera were from well-characterized reference sets that have been widely used for establishing the specificity of serological diagnostic tests [9, 10]. Thirty-two goat antisera against different human influenza A and B strain viral proteins (hemagglutinin, neuraminidase, matrix protein and ribonucleoprotein) were used as additional specificity controls.

**Table 1:** Reference sera used to establish the specificity of COVID-19 IBs

Source	Characteristic	Number of sera
IGeneX (human sera)	Leftover, decoded, pre-pandemic sera received for tick-borne diseases testing	152
CAP and NYSHD Autoimmunity and Allergy (human sera)	Anti-nuclear antibody positive	5
	Anti-dsDNA antibody positive	2
	Rheumatoid factor positive	12
	Rheumatoid factor negative	7
	Elevated IgG	13
	Elevated IgE	4
	Normal IgE	2
NYB Viral Infections (human sera)	Epstein-Barr virus infection	7
	Herpes simplex virus infection	4
	Cytomegalovirus infection	4
	Hepatitis C infection	5
	HIV infection	7
NIH AIDS Reagent Program (human sera)	HIV infection	3
BEI Resources (human sera)	Respiratory syncytial virus infection	4
BEI Resources (goat antisera)	Goat antisera to human influenza A virus proteins	27
	Goat antisera to human influenza B virus proteins	5

*CAP* College of American Pathologists; *NYB* New York Biologics; *NYSH* New York State Department of Health; *NIH* National Institutes of Health; *BEI* Biodefense and Emerging Infections Research Resources Repository; *ds* double stranded

### Sera from COVID-19 patients

Decoded leftover sera received for routine testing at the Medical Art Center, Middletown, NJ and IGeneX, Milpitas, CA that would otherwise have been discarded were used in the study. The 84 sera were from 37 patients who had tested positive by the FDA EUA-authorized Quest Diagnostics RC SARS-CoV-2, LabCorp COVID-19 or ThermoFisher Scientific TaqPath COVID-19 RT-qPCR tests. All patients had only shown mild symptoms of COVID-19 and none had required hospitalization. The 84 serum samples utilized in the study were collected at times that ranged from 0 to 154 days after the patients had tested positive in RT-qPCR tests. The patients were 19 males and 18 females with an age range of 21 to 76 years.

### SARS-CoV-2 protein antigens for Immunoblots

The N protein (amino acid residues 1–419, Genbank accession QHD43423.2), the spike protein (Genbank accession QHD43416.1) S1 domain composed of amino acid residues 16–690, the S2 domain (amino acid residues 698–1213), and the RBD region of the S1 (amino acid residues 319–541), were used as target antigens in the IBs. Protein BLAST analysis (https://blast.ncbi.nlm.nih.gov/Blast) showed that the N and S proteins used in the IBs had ≥99.76% and ≥ 98.84% sequence homologies respectively to the corresponding protein sequences from other SARS-CoV-2 isolates deposited in the GenBank database (taxid 694009). The N and S proteins of SARS-CoV-2 used in IBs had lower sequence homologies of ≤34% and ≤ 33% to the respective N and S proteins from four human coronaviruses (strains OC43, HKU1, 229E and NL63) that cause common cold symptoms [6]. All recombinant antigens for IBs were prepared by cloning relevant portions of the SARS-CoV-2 genes into pET vectors and expressing the proteins in *Escherichia coli* (GenScript, Piscataway, NJ). The recombinant proteins were then extensively purified by metal affinity chromatography and gel filtration and determined to be free from *E. coli* proteins by Coomassie blue staining after SDS-PAGE.

### Antigen strips for COVID-19 IB assays

Antigen strips were prepared essentially as described for our previously developed IB assays for borreliosis [9, 10]. Purified antigens diluted to yield approximately 7–19 ng of protein as a line in each 3 mm strip of membrane were sprayed in straight lines onto nitrocellulose membrane (Amersham Protran, GE Healthcare Life Science, Chicago, IL) using a BioDot liquid dispenser (BioDot, Irvine, CA). Human IgG and IgM (Sigma, St. Louis, MO) were applied as controls C1 and C4 respectively in all IB strips for establishing the specificity of antibody class detection and for confirming the addition of alkaline phosphatase conjugated anti-human antibodies. Protein L (Sigma, St. Louis, MO) was used as control C2 for detecting the addition of human serum. A calibration standard C3 was applied on the test strip for use in all IB assays. The membranes were then blocked with 5% dried skim milk and sliced into 3 mm wide strips. The membrane strips containing antigens could be stored for at least 6 months at 2-8 °C before their use for IB assays.

### Detection of IgG and IgM antibodies in COVID-19 patient sera

IgG and IgM antibodies were detected in the COVID-19 IBs essentially as described for borreliosis IBs [9, 10]. Prior to use, each 3 mm strip was soaked in 1 ml of diluent (100 mM Tris, 0.9% NaCl, 0.1% Tween-20 and 1% dried skim milk) for 5 min in a trough. A 10 μL aliquot of the test or control serum for the IgG IB and 20 μL for the IgM IB, was then added to an IB strip, incubated at ambient temperature for 1 h, and then washed three times with wash buffer (KPL, Gaithersburg, MD). After aspirating the final wash solution, strips for detecting IgG and IgM were incubated with alkaline phosphatase-conjugated goat anti-human IgG at 1:10,000 dilution and goat anti-human IgM at 1:3000 dilution respectively (KPL, Gaithersburg, MD) for 1 h at ambient temperature. After three washes, bands were visualized by reaction with 5-bromo-4-chloro-3-indolyl phosphate nitro-blue tetrazolium (KPL, Gaithersburg, MD). The reactions were terminated by washing with distilled water when the calibration standard C3 produced a visible band. Antigen-reactive bands of lower intensity than the calibration standard were considered negative.

### Statistical calculations

Fisher’s exact test was performed online at https://www.socscistatistics.com/tests/fisher/default2.aspx to determine differences in the proportions of antibodies that reacted with S1, S2, N and RBD proteins. Clinical diagnostic parameters were estimated online at http://vassarstats.net/vsclin.html.

## Results

### Criteria for determining overall positive IgG and IgM antibody responses in COVID-19 IB assays

The reactivity of the 231 reference human sera and 32 goat antisera expected to be negative for antibodies to SARS-CoV-2 proteins (Table 1) and the 84 sera from COVID-19 patients were tested in COVID-19 IgG and IgM IBs and their reactivity with different combinations of antigens in the two IBs analyzed. Recognition of any one of the following combination of two proteins, (i) S1 or S2 and N, (ii) S1 or S2 and RBD, and (iii) N and RBD, in IgG COVID-19 IBs and reaction with any two of the proteins S1, S2, RBD and N in IgM COVID-19 IBs respectively gave optimal specificities for detecting IgG and IgM antibodies in the two IBs. Using these criteria, only four of the 263 reference sera expected to be negative were positive for antibodies to SARS-CoV-2: (i) an IGeneX pre-pandemic era human sera showed an overall positive reaction for IgG antibodies and another for IgM antibodies; (ii) a serum from the autoimmunity reference panel with elevated IgG gave a positive reaction for IgG antibodies and another with rheumatoid factor for IgM antibodies.

### IgG and IgM COVID-19 IB assays specifically detect IgG and IgM classes of antibodies respectively

**Fig. 1:**
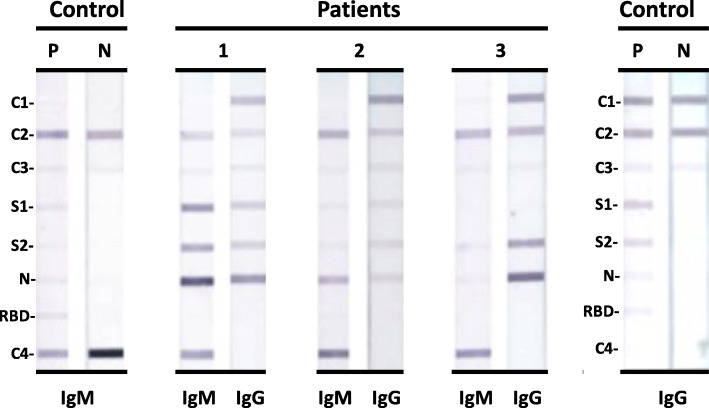
Photographs of COVID-19 IgG and IgM IBs with sera from the three patients (1,2 and 3) and positive (P) and negative (N) control human sera. The positive control (P) was a pool of sera from SARS-CoV-2 RT-qPCR positive patients that reacted with S1, S2, N and RBD proteins in both COVID-19 IgG and IgM IBs. The negative control (N) was pooled human sera from the pre-pandemic period and did not react with the four SARS-CoV-2 antigens. C1: purified IgG; C2: Protein L; C3: internal calibrator; C4: purified IgM. The positions of S1, S2, N and RBD proteins in the membrane strips are also shown

Representative IBs with sera from three SARS-CoV-2 RT-qPCR positive patients and control sera are shown in Fig. 1. The three patient sera shown had both IgG and IgM antibodies reacting with S1, S2 and N proteins. The three sera are classified as positive overall for IgG and IgM antibodies by the criteria described in the previous section. The IBs demonstrate there is no cross-recognition of human IgG and IgM with the two goat antisera to human IgG and IgM used in the IB assays i.e. the IgG IBs only detect IgG antibodies and the IgM IBs detect only IgM antibodies.

### Antibody detection in patient sera varies with time of obtaining sera after a positive RT-qPCR test

Based on the criteria for antibody positivity described above, all but one of the 37 COVID-19 patients developed antibodies that were detected in either COVID-19 IgG or IgM IBs in one or more serum samples provided at different times after the RT-qPCR test. Antibodies were not detected in the sera of only one patient who had provided serum samples on days 15, 30 and 44 after the RT-qPCR diagnosis. The results with the 84 sera from all 37 patients showed that the proportion of sera possessing either IgG or IgM antibodies varied with the time of serum collection after RT-qPCR diagnosis of infection, being 100% for sera collected on days 0–10 and 51–65 after the RT-qPCR test and varying between 92 to 44% for other time periods (Table 2). The results also show that (i) overall IgM antibody positivity was highest in sera obtained 0–10 days after the RT-qPCR test and IgM became negative after 100 days, (ii) overall IgG positivity was 50% in day 0–10 sera after the RT-qPCR test, increased in subsequent periods to reach 100% in the eight sera collected between days 51–65 and then decreased to 44% in sera collected between days 100 to 154. The results with the 37 patients show that the sensitivity of detecting antibodies varied with time after a diagnosis of COVID-19 with positive RT-qPCR test. Sensitivity was 100% in sera collected from days 0 to 10 if both IgG and IgM antibodies are used for diagnosis and also 100% in sera obtained between days 51 to 65 if only IgG antibodies are used.

**Table 2:** Temporal variation of overall IgG and IgM antibody positive responses in patient sera

Days after positive RT-qPCR	Total number of sera tested	No. of sera with IgM antibodies	No. of sera with IgG antibodies	No. of sera with either IgM and/or IgG antibodies
0–10	6	5	3	6
11–20	12	8	11	11
21–30	22	12	18	20
31–40	18	6	15	15
41–50	9	2	7	7
51–65	8	3	8	8
100–154	9	0	4	4

### Variations in the recognition of S1, S2, N and RBD by antibodies in COVID-19 patients

**Fig. 2:**
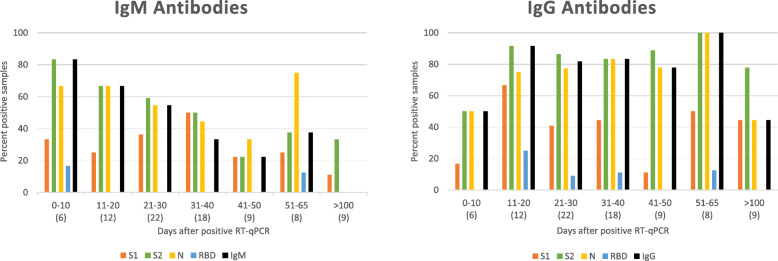
Graphs summarizing IgM and IgG antibody reactivity with the four antigens in IBs with patient sera obtained at different time intervals after a positive RT-qPCR test. The two graphs show the percentage of sera reacting with each of the four test antigens at different time intervals after the RT-qPCR test. The number of sera tested for each time interval are shown in parentheses in the abscissa. Reactivity with the four SARS-CoV-2 proteins are shown in different colors. The black bars show the percentage of sera scored as positive overall for IgM and IgG antibodies using the criteria described above

We also analysed the COVID-IB detectable presence of IgG and IgM antibodies to each of the four test antigens individually in the 84 sera obtained from the 37 COVID-19 patients at varying time intervals after a positive RT-qPCR test. The results are presented graphically in Fig. 2 together with the overall IgG and IgM positivity at each time interval for comparison.

The results in Fig. 2 show that IgG and IgM antibodies to S2 and N were more common than antibodies to S1 and RBD, reaching 100% with S2 and N for IgG antibodies in the eight sera obtained between days 51 to 65. RBD was least frequently detected among the four antigens used in the IBs, with RBD-reactivity ranging from 0–17% for IgM and 0–25% for IgG antibodies in the different time periods. Of the 37 COVID-19 patients, either IgM or IgG antibodies were found reacting in the COVID-19 IBs with each of the proteins S1, S2, N and RBD in 23, 35, 33 and 7 patients respectively. Fisher’s exact test showed that the proportions of either IgM or IgG antibodies in patients that detected S2 and N were signficantly greater than S1 at *p* = 0.001 and *p* = 0.013 respectively and the proportions of either IgM or IgG antibodies detecting S1, S2 and N were significantly more than RBD at *p* < 0.0003. The proportions of patients developing either IgM or IgG antibodies reacting with S2 and N were not significantly different at *p* = 0.67.

### Estimation of clinical parameters of detecting antibodies in COVID-19 IBs

The 231 pre-pandemic human sera that were expected to be negative for antibodies to SARS-CoV-2 and sera from the 37 patients who had COVID-19 infection confirmed by RT-qPCR were used to estimate the sensitivity, specificity and positive and negative predictive values of COVID-19 IBs. We combined findings from all the sera from each COVID-19 patient for these calculations because (i) both IgM and IgG antibody detection in the IBs changed with different samples of the sera that were provided at varying times after the RT-qPCR positive test; (ii) serum samples obtained at different times from the same patient are not independent samples. The results are summarized in Table 3 with details of the calculations shown in Supplementary file S1. They suggest that the sensitivity of detection of IgM antibodies alone in COVID-19 IBs estimated in this way is 70.3%,which is lower than IgG antibodies alone at 91.9%. The estimated sensitivity increases to 97.2% if the sample was considered positive when IgM and/or IgG antibodies were detected. The results suggest that the estimated specificities of detecting antibodies in COVID-IBs estimated in this manner are > 98.2%. The similarly estimated positive predictive values (PPVs) are ≥90% and negative predictive values (NPVs) > 95.4% (Table 3 and Supplementary file S1). COVID-19 prevalence in samples of human sera was estimated to be 37/268 or 13.8% for calculating PPV and NPV (Supplementary file S1).

**Table 3:** Estimated clinical diagnostic parameters of the COVID-19 IB assays

	Estimated	95% Confidence Interval	
	Value	Lower Limit	Upper Limit
**IgM antibodies only**			
**Sensitivity**	0.7027	0.5283	0.8356
**Specificity**	0.9913	0.9657	0.9985
**PPV**	0.9286	0.7504	0.9875
**NPV**	0.9542	0.9172	0.9757
**IgG antibodies only**			
**Sensitivity**	0.9189	0.7698	0.9788
**Specificity**	0.9913	0.9657	0.9985
**PPV**	0.9444	0.7999	0.9903
**NPV**	0.9871	0.9596	0.9967
**IgG and/or IgM antibodies**			
**Sensitivity**	0.9723	0.8419	0.9986
**Specificity**	0.9827	0.9533	0.9944
**PPV**	0.9000	0.7540	0.9675
**NPV**	0.9956	0.9720	0.9998

*PPV* positive predictive vale; *NPV* negative predictive value

## Discussion

The clinical sensitivity and specificity of the COVID-19 IB assays for IgM, IgG and IgG and/or IgM antibodies, estimated by pooling multiple samples from the same patient, meet the US recommendations for laboratory serological diagnostic tests [11]. They fall short of the expected specificity of > 99.5% in the US for large sero-surveillance studies in the community where the prevalence is expected to be very low [3]. However, it is possible that COVID-19 IBs may be useful for sero-epidemiological studies in specific populations with a high prevalence of COVID-19. Our results confirm other common observations in COVID-19 that IgM and IgG antibody levels vary with time after infection [3, 4, 11]. This is expected because antibodies are produced with a lag period after infection, early IgM is later replaced with IgG antibodies and antibody levels in blood generally decrease with time after resolution of infection. Our findings suggest that determining both IgG and IgM antibodies early in an infection i.e. before about 10 days from onset of disease, and IgG antibodies later at about 8 weeks after infection, provide the best sensitivity for detecting antibody responses in COVID-19 IBs. However, more extensive testing of patient samples and other human sera as specificity controls will better establish the clinical diagnostic parameters of the COVID-19 IBs and the optimal times for their use after infection.

Results using pre-prepared IB membrane strips can be obtained in less than 3 h after serum or plasma collection, with minimal washing and reagent addition steps. Also, the assay yields visible signals that are stable for several weeks and readily interpreted relative to an internal calibrator. Furthermore, the IB assay can be adapted for detecting antibodies of other immunoglobulin classes, and in other relevant fluids, e.g. saliva and tears, which is important because the mucosal IgA and blood IgG and IgM antibody responses can differ significantly in COVID-19 [12, 13]. The COVID-19 IB assay can also be easily expanded to include additional virus antigens. Fixed dilutions of patient sera (1:100 in the IgG and 1:50 in the IgM assays respectively) were used to obtain the present results. Dilutions of sera however can be readily varied, together with relevant specificity controls, to generate antibody titers from the COVID-19 IB assays.

The criteria for antibody positivity utilized the necessary recognition of at least two of the four SARS-CoV-2 proteins for optimizing the specificity of COVID-19 IgG and IgM IB assays. The RBD lies within the S1 region of the S protein but the detection of RBD by antibodies did not parallel the detection of S1, with RBD being detected by fewer sera and variably at different time periods compared to S1. Epitopes in regions other than the RBD in S1 are therefore importantly antigenic in patients. Antibodies to the RBD in particular and the more N terminal region of S1 are important for neutralizing virus infectivity by preventing binding to host cells [14, 15]. Some antibodies to S2 may also neutralize infectivity by inhibiting cell fusion and virus entry [16, 17]. Measurement of antibody titers in the IB assay may be relevant as IgG antibody titers to the S protein measured by ELISA correlate with virus-neutralizing antibody titers in persons vaccinated with S [8], although this correlation is weaker in non-hospitalized patients [18]. Other data suggest that antibody levels to RBD and other viral antigens are higher in more severely ill hospitalized patients [19, 20], which may be consistent with the weaker anti-RBD antibody responses in non-hospitalized patients seen in the present study.

One of the 37 patients did not show detectable antibodies when tested on days 15, 30 and 44, a phenomenon which has previously been observed in other non-hospitalized patients [18]. It is possible that this patient’s sera might have possessed detectable levels of antibodies had it been tested prior to day 10 or between days 51 to 65, periods after infection when the overall sensitivity of detecting antibodies was 100% in the COVID-19 IB assays. The decline in antibodies in the 100 to 154 day period after a positive RT-qPCR test may be partly characteristic of the relatively mild disease studied here since antibody levels are reported to be more sustained in severely ill patients [21]. The findings emphasize the importance of detecting both IgG and IgM antibodies rather than either antibody class alone, and at different times after infection, for assessing seroconversion in COVID-19, which is consistent with the findings in symptomatic patients from China [20].

## Conclusions

The COVID-19 IB assays offer advantages that make them useful additions to existing serological tests for confirming active or past infection and assessing antibody responses in patients with active disease.

## Supplementary Information

**Additional file 1.** provides details of the calculations of the estimated clinical parameters of the detection of IgG and IgM antibodies in COVID-19 immunoblots.

## Data Availability

The datasets generated and/or analyzed during the current study, unlinked to patient identities, are available from the corresponding authors on reasonable request.

## Abbreviations

BLAST: Basic Local Alignment Search Tool
COVID-19: Coronavirus Disease 2019
EIA: Enzyme Immunoassay
IB: Immunoblot
N: Nucleocapsid protein of SARS-CoV-2
NAAT: Nucleic acid amplification test
RBD: Receptor binding domain of spike protein
RT-qPCR: Quantitative reverse transcription polymerase chain reaction
SARS-CoV-2: Severe acute respiratory syndrome coronavirus 2
S: Spike protein of SARS-CoV-2
